# Mental health and human rights of women

**DOI:** 10.1192/j.eurpsy.2021.150

**Published:** 2021-08-13

**Authors:** M. Amering

**Affiliations:** Department of Psychiatry and Psychotherapy, Medical University of Vienna, Vienna, Austria

**Keywords:** womens’ rights, human rights, non-discrimination, advocacy

## Abstract

**Introduction:**

Mental health stigma and discrimination interact with gender inequality and the discrimination of women and girls to their mental health detriment.

**Objectives:**

Present and discuss the challenges and opportunities of a human rights based approach to womens’ mental health.

**Methods:**

Non-systematic review of policy and practice of human rights based interventions for womens’ mental health.

**Results:**

Current mental health as well as gender equality legislation converge towards the realization of longstanding demands of equality for women as well as for persons with mental health problems: removal of barriers, respect and enablement of autonomy, renewed efforts toward effective inclusion in all spheres of life. Essential changes through non-discrimination laws concern key areas, including family planning, marriage and parenthood, employment, housing, education, health, standards of living and social, political and cultural participation, along with the right to be free from exploitation, violence and abuse. Because of the cumulation and the interaction of gender-based and other forms of discrimination, legislations such as the UN-Convention on the Rights of Persons with Disabilities (UN-CRPD) include a focus on gender-specific human rights needs of women and girls. Family advocacy in mental health is prominently supported by female activists as is the user movement.

**Conclusions:**

The opportunities of a successful development towards non-discrimination and gender equality in mental health care are dependent on a viable understanding of these concepts within the mental health community as well as updated expertise concerning tools for implementation of support systems sensitive to the human rights needs of women and girls.

**Disclosure:**

No significant relationships.

